# Proteomics Unveils Post-Mortem Changes in Beef Muscle Proteins and Provides Insight into Variations in Meat Quality Traits of Crossbred Young Steers and Heifers Raised in Feedlot

**DOI:** 10.3390/ijms232012259

**Published:** 2022-10-14

**Authors:** Mariane Severino, Mohammed Gagaoua, Welder Baldassini, Richard Ribeiro, Juliana Torrecilhas, Guilherme Pereira, Rogério Curi, Luis Artur Chardulo, Pedro Padilha, Otávio Machado Neto

**Affiliations:** 1 College of Agriculture and Veterinary Science (FCAV), São Paulo State University (UNESP), Jaboticabal, Sao Paulo 14884-900, Brazil; 2 Food Quality and Sensory Science Department, Teagasc Food Research Centre, Ashtown, Dublin 15, D15 DY05 Dublin, Ireland; 3 Physiologie, Environnement et Génétique Pour l’Animal et les Systèmes d’Élevage (PEGASE), INRAE, Institut Agro, 35590 Saint-Gilles, France; 4 College of Veterinary Medicine and Animal Science (FMVZ), São Paulo State University (UNESP), Botucatu, Sao Paulo 18618-681, Brazil; 5 Institute of Bioscience (IB), São Paulo State University (UNESP), Botucatu, Sao Paulo 18618-681, Brazil

**Keywords:** beef quality, proteome, carcass properties, gender, mass spectrometry, 2D-PAGE

## Abstract

Proteomics has been widely used to study muscle biology and meat quality traits from different species including beef. Beef proteomics studies allow a better understanding of the biological processes related to meat quality trait determination. This study aimed to decipher by means of two-dimensional electrophoresis (2D-PAGE), mass spectrometry and bioinformatics the changes in post-mortem muscle with a focus on proteins differentially expressed in the *Longissimus thoracis* (LT) muscle of immunocastrated young heifers and steers. Carcass traits, chemical composition, pH, instrumental color (L*, a*, b*), cooking loss and Warner-Bratzler shear force (WBSF) of meat from F1 Montana-Nellore cattle were also evaluated. Backfat thickness (BFT) and intramuscular fat content (IMF) were 46.8% and 63.6% higher in heifers (*p* < 0.05), respectively, while evaporation losses (EL) were 10.22% lower compared to steers. No differences (*p* > 0.05) were observed for tenderness evaluated by WBSF (3, 10, and 17 days post-mortem), pH, and color traits (L*, a* and b*) between the experimental groups. The study revealed several proteins to be differentially expressed proteins in heifers compared steers (*p* < 0.05). In heifers, proteins involved in nutrient transport (TF, ALB, and MB), energy metabolism (ALDOA, GAPDH, and PKM), and oxidative stress and response to stress (HSPA8 and CA3) were associated with a greater BFT and IMF deposition. The higher expression of these proteins indicated greater oxidative capacity and lower glycolytic activity in the LT muscle of heifers. In steers, there was greater abundance of protein expression related to muscle contraction and proteins of structure (ACTA1, TPM2 and TNNT3), energy metabolism (ENO1, ENO3, PYGM, PGM1 and TPI1) and ATP metabolism (ATP5F1B, PEBP1 and AK1), indicating greater glycogenolysis in LT muscle, suggesting a shift in the glycolytic/oxidative fibers of steers.

## 1. Introduction

Gender or the sexual condition of cattle is known to be an important factor affecting animal performances and growth, carcass properties and meat quality [1]. Such differences are related to tissue growth as well as their distribution in the carcass. Likewise, castration can improve body fat deposition in beef cattle, and limited sexual behavior leads to easier rearing practices and less carcass damage, which improves carcass fatness and, consequently, meat quality traits [2]. Alternatively, immunological castration (immunocastration) is a relatively new approach, and some studies have described the effects on meat quality of beef cattle [3,4]. However, there is scarcity in the studies comparing young heifers and steers produced according to the “super early-maturing” system that allocate animals to feedlot feeding at 8 months of age (slaughtered up to 15 months of age, [5]). Although immunocastration of female cattle is not a common practice in tropical beef production systems like Brazil, it has the potential to be an easily applied tool to improve the welfare of heifers and cows used for beef production, as reviewed [6]. Moreover, no studies have reported so far reported the relationships between meat quality traits and muscle tissue proteome and which pathways are impacted in crossbred young heifers and steers immunocastrated and feedlot finished.

Proteomics is a powerful tool in meat science due to its technical performance in understanding the effect of the factors influencing meat quality as well as in deciphering the biochemical and metabolic mechanisms occurring to muscle-to-meat conversion in the post-mortem period [7,8,9,10]. It further allows for the study each quality trait, such as pH decline, muscle proteins degradation during ageing, oxidation and post-translational modification of proteins in an in-depth manner [11,12,13]. Thus, omics-related analytical technologies and bioinformatics tools have been significantly applied in the last two decades in the field of meat research to identify proteins related to several meat quality traits, since they are the main constituents of muscle tissue and are responsible for the regulation of main metabolic pathways [11]. In this context, meat quality research in beef cattle and other livestock species used the proteomic approach to evaluate tenderness [7,14,15], marbling [10], color [16,17], water-holding capacity [18,19], and dark-cutting beef [20] among other meat quality traits [11].

The hypothesis of the present study was that differences might exist in the carcass properties and meat quality traits of heifers and steers, particularly the deposition of subcutaneous and intramuscular fat, which can be a consequence of protein changes and their expression. In this context, the aim of this study using a proteomics approach was to identify proteins differentially expressed in the *Longissimus thoracis* (LT) muscle, and how they are related with the physicochemical differences of meat produced by immunocastrated F1 Montana-Nellore young heifers and steers feedlot finished. We further aimed to reveal the molecular pathways and mechanisms behind such mechanisms using advanced bioinformatics analyses.

## 2. Results

### 2.1. Carcass Traits, Chemical Composition and Meat Quality

Backfat thickness (BFT) of heifers’ carcasses was 46.8% higher (*p* < 0.05) compared to steers’ (Table 1). The variables FBW and CY were not altered between genders, while HCW and REA tended to be greater in steers (*p* < 0.10). Intramuscular fat (IMF) content of heifers was 63.6% higher and moisture content was 2.22% lower than in steers (Table S1). Evaporation loss (EL) was 10.22% higher in steers. On the other hand, DL was 22.3% higher in heifers, while CL, WBSF, pH and lightness (L*) were influenced by the ageing period (Table 2).

### 2.2. Muscle Tissue Proteome

**Figure 1:**
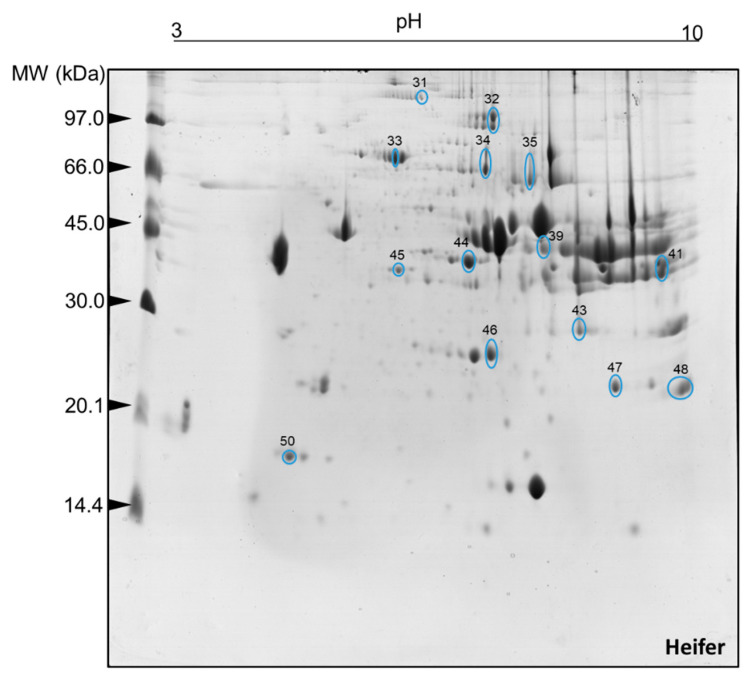
Protein spots selected for characterization by mass spectrometry (ESI-MS) after image analysis. Two-dimensional polyacrylamide gel electrophoresis (2D-PAGE): 12.5% (*w*/*v*) and pH gradient 3–10. Muscle tissue samples (*Longissimus thoracis*) from immunocastrated F1 Montana-Nellore young heifers feedlot finished. The information on the number IDs of differentially abundant spot proteins are given in Table 3.

**Figure 2:**
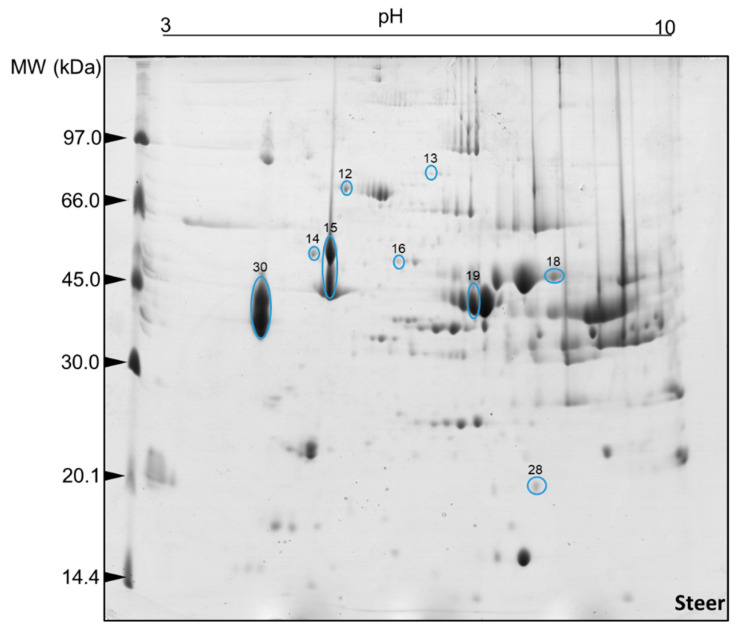
Protein spots selected for characterization by mass spectrometry (ESI-MS) after image analysis. Two-dimensional polyacrylamide gel electrophoresis (2D-PAGE): 12.5% (*w/v*) and pH gradient 3–10. Muscle tissue samples (*Longissimus thoracis*) from immunocastrated F1 Montana-Nellore young steers feedlot finished. The information on the number IDs of differentially abundant spot proteins are given in Table 3.

The comparison of the 2DE gels allowed us to see that the mean number of protein spots per treatment was 119 ± 25 for steers and 115 ± 32 for heifers. The number of spots of the reference gels were 150 for steers and 160 for heifers. Of this total, after the imaging analysis investigation, 50 spots were selected as differentially expressed (*p* < 0.05) in heifers (Figure 1) and steers (Figure 2).

Several proteins were identified that were abundantly expressed in the muscle of steers and heifers (Table 3). The proteins identified perform functions of muscle contraction/regulation, carbohydrate metabolism, ATP activity, cytoprotection, cellular defense, energy metabolism, and binding. Three main groups (clusters) were distinguished in heifers: oxidative stress and cell defense proteins (HSPA8 and CA3), proteins related to muscle contraction (MYBPC1, TNNT1 and TNNI2), and energy metabolism proteins (CKM, PKM, ALDOA and GAPDH). Regarding the proteins identified in steers, there were groups (clusters) of proteins related to energy metabolism (ATPIF1, ENO1, ENO3, PEBP1, PYGM, PGM1 and TPI1) and muscle contraction (TPM2, TNNT3 and ACTA1).

**Table 3:** Proteins separated by two-dimensional electrophoresis (2D-PAGE) and identified by mass spectrometry (ESI-MS/MS) in samples of *Longissimus thoracis* muscle from immunocastrated F1 Nellore-Montana young heifers and steers feedlot finished.

Spot ID	Gene Symbol	Full Protein Names	Uniprot ID	Mascot Score	Coverage (%)	p*I*/MW Experimental	p*I*/MW Theoretical	Expression ^1^
Energy metabolism								
16	ENO1	Phosphopyruvate hydratase	A0A3Q1MXQ0	5261.196	47.00	6.17/49,703	6.48/54,808	1.95 (UP in steers)
18	ENO3	Beta-enolase	Q3ZC09	10,199.91	57.60	8.19/44,261	7.55/47,438	5.51 (UP in steers)
32	PYGM	Alpha 1–4 glucan phosphorylase	F1MJ28	7318.068	63.42	7.09/97,750	7.05/97,735	1.13 (UP in steers)
34	PGM1	Phosphoglucomutase-1	Q08DP0	9944.941	78.11	6.99/65,655	7.21/61,874	1.89 (UP in steers)
46	TPI1	Triosephosphate isomerase	Q5E956	21,146.33	88.35	7.29/25,236	7.27/26,917	1.28 (UP in steers)
47	AK1	Adenylate kinase isoenzyme 1	P00570	12,755.28	58.76	8.71/22,375	8.35/21,778	1.73 (UP in steers)
35	PKM	Pyruvate kinase	A5D984	12,176.24	63.09	7.7/60,091	7.1/58,519	1.37 (UP in heifers)
39	ALDOA	Fructose-bisphosphate aldolase	A6QLL8	7590.983	70.33	7.73/42,863	7.94/39,949	2.20 (UP in heifers)
41	GAPDH	Glyceraldehyde-3-phosphate dehydrogenase	P10096	30,163.61	47.75	9.19/34,988	8.97/36,096	4.01 (UP in heifers)
19	CKM	Creatine kinase M-type	Q9XSC6	16,598.07	70.60	7.02/43,080	6.8/43,217	1.89 (UP in heifers)
14	ATP5F1B	ATP synthase subunit beta, mitochondrial	P00829	12,307.51	56.44	5.05/51,780	5.04/56,283	2.07 (UP in steers)
Muscle structure								
31	MYBPC1	Myosin binding protein C1	A6QP89	1502.969	33.97	6.23/113,152	6.5/135,007	4.07 (UP in heifers)
30	TPM2	Tropomyosin beta chain	Q5KR48	5317.557	44.01	4.33/40,213	4.55/32,950	1.44 (UP in steers)
45	TNNT1	Troponin T, slow skeletal muscle	Q8MKH6	3035.667	19.39	5.88/37,343	5.67/31,284	2.49 (UP in heifers)
48	TNNI2	Troponin I2, fast skeletal muscle	F6QIC1	3650.82	54.49	9.44/21,323	9.78/21,141	2.66 (UP in heifers)
44	TNNT3	Troponin T, fast skeletal muscle	Q8MKI4	1410.723	36.9	6.84/39,524	6.85/32,124	1.37 (UP in steers)
15	ACTA1	Actin, alpha skeletal muscle	P68138	29,409.83	81.96	5.28/42,998	5.23/42,393	2.50 (UP in steers)
Binding proteins								
13	TF	Serotransferrin	Q29443	270.5198	17.19	6.58/82,905	6.6/79,920	1.74 (UP in heifers)
33	ALB	Albumin	P02769	13261.69	64.91	5.97/69,477	5.87/71289	1.19 (UP in heifers)
50	MB	Myoglobin	A0A1K0FUF3	11,507.29	56.49	4.49/17,121	7.80/17077	3.65 (UP in heifers)
28	PEBP1	Phosphatidylethanolamine binding protein 1	P13696	6676.657	64.17	7.94/21,444	7.82/21099	2.08 (UP in steers)
43	CA3	Carbonic Anhydrase 3	Q3SZX4	10,936.17	60.00	8.37/27,806	8.1/29655	5.24 (UP in heifers)
Heat shock proteins								
12	HSPA8	Heat shock cognate 71 kDa protein	P19120	19,088.86	44.92	5.46/73,533	5.25/71468	2.59 (UP in heifers)

^1^ Protein spots expression in experimental groups (steers versus heifers). For 2D gel image comparisons, a reference gel per treatment was listed, which contained the highest number and best definition of spots, and the reference gel of a treatment was contrasted with each gel of another treatment, totaling 15 comparisons.The number of BP, MF and CC of the identified proteins differed between heifers (Figure 3) and steers (Figure 4). Considering the distribution of the top 20 levels (Gene Ontology), BPs were identified in greater quantities in steers, among which cellular metabolism (19), nitrogen compounds (16), small molecules and biosynthesis (16) stand out. Divergent processes were observed in smaller amounts in heifers, the main ones being related to establishment (8), vesicle-mediated transport (6) and response to oxidative stress (5). Regarding MF, a predominance of membrane transport (14) and ionic binding (8) proteins was observed in steers. Ionic binding sequences (5) were also verified in heifers, as well as with hydrolysis activity (3). The CC identified in both treatments was similar between the groups, with emphasis on several proteins associated with the organization of cell structure, cytoplasmic and those present in organelles.

**Figure 3:**
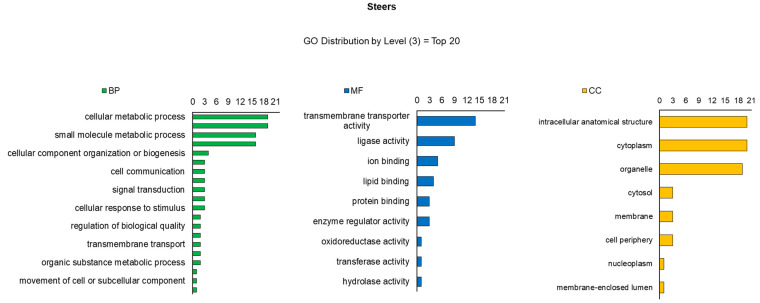
Classification of proteins identified in tissue samples (*Longissimus thoracis*) from immunocastrated F1 Montana-Nellore young heifers feedlot finished. The OMICSBOX software was used to classify the proteins according to biological process (BP), molecular function (MF), and cellular component (CC).

**Figure 5:**
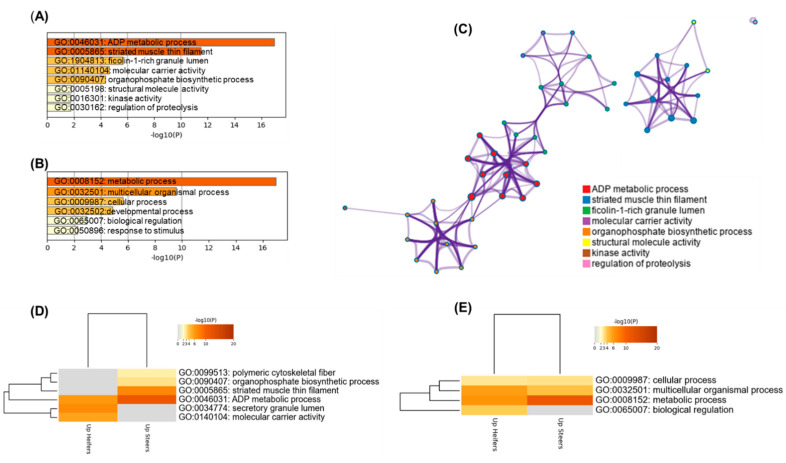
Bioinformatics analyses based on the differentially expressed proteins identified in this experiment. (**A**) Enriched ontology clusters based on the significantly enriched gene ontology (GO) terms obtained using the protein lists of steers (*n* = 11) and heifers (*n* = 12) identified in *Longissimus thoracis* muscle tissue of feedlot-finished immunocastrated F1 Montana-Nellore. (**B**) Enriched biological process. The graphs highlight all the enriched terms across the protein lists with the importance of energy metabolism (metabolic and ADP processes), metabolic, cellular and developmental processes according to −Log *p*-values. (**C**) Enriched network related to the previous terms of (**A**) highlighting the degree of interconnectedness. (**D**,**E**) Hierarchical heatmap clustering comparing the enriched GO terms within steers and heifers as well the main biological process in each condition. The heatmaps colored by the *p*-values are indicated by color, where grey cells indicate the lack of significant enrichment, palest brown indicates a low *p*-value and darkest brown indicates a high *p*-value.

The main enriched terms and pathways identified in this study using the differentially expressed proteins for heifers and steers are summarized in Figure 5. Based on gene ontology terms, the ADP metabolic process through “striated muscle thin filament” was highly and significantly up-regulated in steers compared to heifers. Cluster pathways related with cellular and developmental processes were more enriched in heifers. Such pathways of generation of metabolites and energy in heifers help to explain the higher IMF found in the meat of these animals.

**Figure 6:**
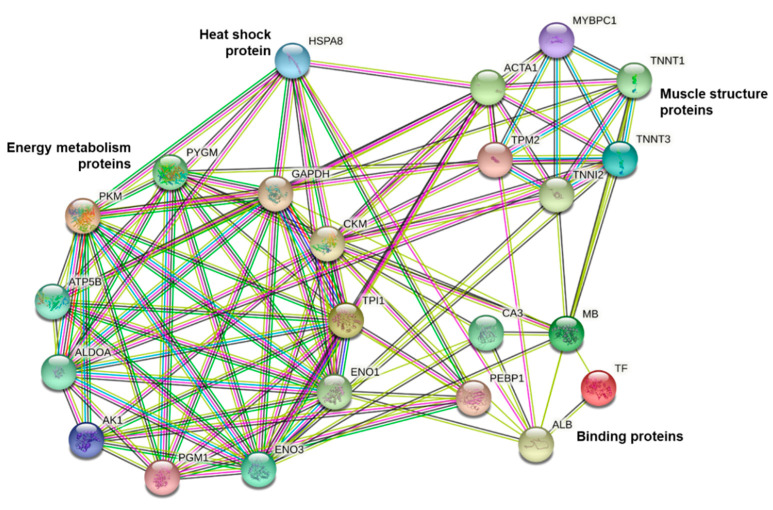
Analysis of protein-protein interactions using the differentially expressed proteins in muscle tissue (*Longissimus thoracis*) of feedlot-finished immunocastrated heifers and steers F1 Montana-Nellore.

The current GO analysis (Figure 5D) suggests that “secretory granule lumen and “molecular carrier activity” are associated specific to heifers and can be associated with greater fatness (BFT in carcass and IMF in meat). Additionally, “biological regulation” was specifically associated with greater IMF found in heifers. Other GO terms were common to both protein lists, some that were more significant for steers such as metabolic process (Figure 5E). The protein-protein interactions were analyzed (Figure 6). There were two main groups (clusters): proteins related to energy metabolism (PKM, PYGM, GAPDH, CKM, TPI1, ENO1, ENO3, PGM1, AK1, ALDOA, and ATPSB) and proteins related to muscle contraction (TNNT1, TNNT3, TNNI2, TPM2, ACTA1 and MYBPC1). Furthermore, a small interaction network involving binding proteins (ALB, TF, MB, PEBP1 and CA3) and heat shock protein (HSPA8) was identified.

Several Quantitative trait loci (QTL) were found to be related with carcass and meat quality traits (*n* = 12). Overall, 10 chromosomes grouped the 21 proteins (gene names) and, among the major QTLs, most of the proteins were related to the energy metabolism pathway, followed by signaling and transport. Several of the proteins found to change in this study were biomarkers of marbling degree, IMF, beef tenderness and QTLs at the same time (Table 4).

## 3. Discussion

In tropical countries, such as Brazil, more than 80% of the beef come from pasture systems distributed over 170 million hectares of land. Feedlot systems are used as an alternative to ensure beef supply mainly during the dry season, with better quality to regional and international markets. According to a recent survey, animals on these feedlots included mostly Nellore (75%), a *Bos indicus* cattle, but also some European × Nellore crossbreeds, and other Zebu breeds [21]. Although Nellore cattle is by far the most prevalent breed in tropical regions of Brazil, breeds like Aberdeen Angus and Bonsmara and, also, composite programs (e.g., Montana Tropical) are increasingly growing in the last decades [22].

This is the first study to report that differences in the meat quality of F1 Nellore-Montana young steers and heifers feedlot finished, are related with the expression of several proteins from different pathways such as ALB, MB, CA3, ALDOA, GAPDH, PKM, PYGM, PGM1, HSPA8, and CKM in the post-mortem LT muscle. Some of these proteins were reported as biomarkers of IMF deposition in other studies [11,23,24,25].

### 3.1. Proteins with Possible Roles in Intramuscular Fat Content and Meat Quality

In the present study, the greater IMF of heifers can be linked with proteins involved in nutrient transport (ALB and MB), energy metabolism (ALDOA, GAPDH, PKM), and cell protection and response to stress (HSPA8). In fact, these proteins are known to be associated with greater IMF deposition capacity, which consequently can be the reason for the generation of lower EL. Proteins differentially expressed in the LT muscle of heifers also revealed the enrichment of the oxidative activity pathways (ALB, MB and CA3 proteins). Moreover, glycolytic/gluconeogenesis enzymes (ALDOA, GAPDH and PKM) may characterize the use of non-carbohydrate substrates in the production of energy by the tissue [26], thus helping to explain the higher proportion of IMF in the meat of immunocastrated heifers when compared to immunocastrated steers. Further studies using shotgun proteomics approaches on a higher number of animals are needed to validate these proteins as potential biomarkers.

Proteins such as ALB and MB indicated oxidative activity in the muscle of heifers. Albumin regulates the colloidal osmotic pressure of the blood, and binds and transports fatty acids, cholesterol and some ions (copper, zinc and calcium) via the bloodstream [27]. A previous study by Baldassini et al. [28], identified the higher expression of ALB in Nellore bulls with higher IMF and lower WBSF (more tender meat). This protein was also described in another study using a similar gel-based proteomic approach [29], whereby ALB was associated with IMF content. The uptake of fatty acids by ALB indicates the use of lipids as muscle oxidative substrate, used in slow-twitch fibers (type I) which can support the oxidative activity in the muscle of heifers. In addition, myoglobin carries the oxygen necessary for oxidative metabolism, a characteristic of slow-twitch (type I) fibers [30,31] in the muscle of heifers.

The proteins ALDOA, GAPDH and PKM are enzymes of the glycolysis and gluconeogenesis pathways. ALDOA reversibly converts fructose-1,6-bisphosphate to glyceraldehyde-3-phosphate and dihydroxyacetone phosphate. Picard et al. [32] reported similar results to the present study for European cattle, in which ALDOA was more expressed in cows compared to castrated males. The enzyme GAPDH also reversibly converts glyceraldehyde 3-phosphate to 1,3-bisphosphoglycerate. In this sense, an earlier study found higher expression of the enzymes GAPDH and ALDOA in the proteome of steers, with higher IMF content, when compared to Nellore bulls [24]. In the present study, these enzymes were also more abundant in heifers due to the higher IMF content in the meat.

The allosteric enzyme PKM catalyzes the irreversible transfer of a phosphate group from phosphoenolpyruvate to an ADP molecule with final production, at the substrate level, of pyruvate and ATP [33]. In the present study, the proteome of heifers indicates greater glucose degradation by the oxidative pathway, suggesting greater mitochondrial activity with greater synthesis of fatty acids for IMF deposition.

Carbonic anhydrase (CA3) has the function of reversible hydration of carbon dioxide (CO_2_) and is closely related to increased oxidative metabolism. In proteomic studies conducted using steers of Asian origin, Qinchuan [34] and Hanwoo [35], CA3 was less expressed in animals with high marbling, leading to divergent results to the present study. The protein CA3 is present in higher concentrations in the cytoplasm of skeletal muscle cells with a predominance of type I fibers, stimulating ATP synthesis by the rapid conversion of glycolytic intermediates into oxaloacetate and citrate [36]. Again, the greater expression of CA3, together with PKM, suggest greater oxidative metabolic activity in the muscle tissue of heifers compared to steers. Although characterization of muscle fibre type was not performed in the current study, further biochemical approaches are needed to validate this hypothesis.

In addition to that, creatine kinase Type M (CKM), converting ADP to ATP from phosphocreatine, provides energy for muscle and reported to affect meat quality and its variability [7,37,38]. The greater expression of CKM in the muscle of heifers may be indicative of a greater energy demand of ATP to maintain muscle functions immediately after slaughter.

The proteins PYGM and PGM1 are key enzymes in the use of glycogen as a substrate for the synthesis of glucose-6-phosphate and glycogenolysis [39]. Researchers reported that PYGM was overabundant in muscle from low growth rate than high growth rate crossbred steers at the time of harvest [29]. After slaughter, this metabolic pathway is extremely important in the anaerobic synthesis of ATP with the final accumulation of intracellular lactate. The greater expression of these proteins in the muscle of steers indicates a greater gluconeogenesis, probably due to the greater accumulation of glycogen ramifications, typical of muscles with a greater number of glycolytic/oxidative fibers (Type IIA) [31,40], fibers with greater hypertrophic capacity, and water accumulation.

The chemical composition of the meat from steers showed higher moisture compared to heifers, which showed a higher amount of IMF. Each gram of glycogen retains four grams of water that is released in the cooking process [41], a fact that could explain the lower water-holding capacity (or higher EL) observed in beef from steers. Moreover, researchers [42] analyzed proteins expression related to both IMF and visceral fat in cows and bulls, and reported that TPM2 expression in adipose tissues were lower in bulls compared to cows, suggesting that TPM2 is positively associated with marbling score and quality grade. Our data also have demonstrated that TPM2 were differentially expressed depending on sex, which indicates that sex hormones are key factors affecting the TPM2 expression and, consequently, lipid accumulation in meat. Therefore, when there is more IMF there is less water content in the heifers’ meat.

### 3.2. Key Roles of Oxidative Stress and Cell Defense

The cognate heat shock protein (HSPA8) has a key role in cellular cell death such as apoptosis and autophagy, conferring greater selectivity to degrading proteins in the lysosome [43]. They are also involved in the cytosolic export of nuclear proteins [43]. This is a well-known biomarker of beef quality (tenderness) from the heat shock proteins pathways revealed by Gagaoua and co-workers in their integromics meta-analysis [7]. The HSP70 was also shown to play a role in osteogenesis by upregulating the expression of osteogenic genes [44]. Therefore, HSP70 could be associated with carcass traits through an involvement in muscle and skeletal development as previously evidenced by Gagaoua et al. [45]. Working with pigs, Di Luca et al. [19] reported the increased abundance of HSPs in samples with low post-mortem muscle exudate. The greater expression of HSPA8 in the muscle of heifers may be indicative of a more effective establishment of *rigor mortis*, suggesting a greater proteolytic efficiency under stress conditions (hypoxia).

### 3.3. Muscle Structure, Contractile and Associated Proteins

In steers, the greater abundance of proteins related to muscle contraction and structure (ACTA1 and TNNT3) allow for the explanation of the results of HCW and REA, which trend to be greater in these animals compared to heifers. Some of these proteins identified have been related to muscle growth in other previous studies [46,47], which agree with the results observed in the current study. Moreover, an earlier proteomic study on feedlot finished lambs [48] reported greater expression of proteins TNNT3 and MYL1 in LT muscle. Both were related to the regulation of myosins and, consequently, muscle growth. Thus, the main factors involved in muscle contraction, as observed in the present study, may be affected by gender status.

Similarly, steers and heifers slaughtered at the same final body weight (FBW) differ in carcass traits and meat quality, as reported in previous studies [1]. Such differences can be related to a greater abundance of protein expression related to muscle contraction and proteins of structure (ACTA1 and TNNT3), tissue growth, as well as carcass fatness. Moreover, as reported in the current study, whereby contractile and associated proteins were highly and significantly up-regulated in steers, researchers [29] found that ACTA1 and TNNT3, involved in biological pathways such as glycolysis/gluconeogenesis and muscle contraction, were upregulated in crossbreed feedlot finished steers, regardless of meat aging period. These authors also reported that the growth rate (feedlot versus pasture finishing systems) affected proteins expression in LT muscle and led to an overabundance of ACTA1 and TNNT3, which are good biomarkers of beef tenderness, as reported in other studies [7,17]. However, in the current study, despite the effects on the LT proteome, tenderness evaluated by WBSF was not affected by gender.

### 3.4. Limitations

The inclusion of one additional biological type in the experimental design of the study, for example, a third group (“control” bulls), would have been an advantage for this study for robust comparisons of differences in skeletal muscle proteome and meat quality traits. Such an experimental group may help to better describe the protein expression in LT muscle to further dissect the contribution of the individual changing proteins belonging to energy metabolism, nutrient transport and signaling pathways, as well as proteins of muscle contraction in response to sexual condition or gender, as reported in the literature [1,2,24].

Although several molecular mechanisms are affected after slaughter according to the literature [49,50], such differences sometimes do not reflect changes in final beef quality, as observed in the current study for tenderness (WBSF), and the meat color of crossbred young heifers and steers raised on feedlot. The WBSF and color variables did not reach statistical significance, which may be due to the small number of animals used for the meat quality. Taken together, these results suggest that a greater number of animals should be evaluated in the future.

## 4. Materials and Methods

### 4.1. Animals, Carcass Traits and Muscle/Meat Sampling

Sixteen-immunocastrated F1 Montana-Nellore animals (eight heifers and eight steers), half siblings, were fattened in an experimental feedlot at of the São Paulo State University “Júlio de Mesquita Filho”–UNESP (Botucatu, São Paulo, Brazil) from December 2018 to April 2019. The animals were housed in collective pens and separated by gender. All animals received the same diet and three doses of the immunocastration vaccine (Bopriva^®^) throughout the finishing period. The first, second and third doses of vaccine were applied at 30, 60 and 90 days after weaning (8 months of age), respectively. The immunocastration of the females was carried out aiming to submit these animals to the same rearing conditions as the males. Additionally, this condition causes temporary immune suppression of ovarian function, reducing estrogen and progesterone levels, thus decreasing ovarian and uterine weights. In addition, this practice was also applied to prevent mounting behaviors and their associated injuries, which help to improve heifer welfare [6].

The diet (% dry matter, DM) was composed of 15% forage (sugarcane bagasse) and 85% concentrate (64% corn, 17% soybean meal, and 4% mineral mixture). Males and females started the feedlot with an initial body weight (BW) of 284.00 ± 45.26 kg and 289.40 ± 17.40 kg, respectively. The animals were weighed at the beginning and at the end of the experimental period, which lasted 110 days. The experimental groups were slaughtered at 15 months of age in a commercial slaughterhouse in the city of Boituva, São Paulo, located 120 km from the place where the experiment was conducted. This followed the state inspection procedures and was preceded by 16-h water and feed fasting. After slaughter, the carcasses were individually weighed to record the hot carcass weight (HCW) and carcass yield (CY), which was calculated using final BW and HCW (CY = HCW/BW × 100). Individual samples (approximately 2 g) of LT muscle were collected from the right half carcass between the 12th and 13th thoracic vertebrae in the hot carcass (pre rigor mortis) and frozen in liquid nitrogen.

Subsequently, the LT samples were stored in a freezer (−80 °C) until they were used in proteomic analyses. The carcasses were cooled for approximately 48 h at 1 °C. During deboning (48 h of cooling), the backfat thickness (BFT) was then measured with a caliper between the 12th and 13th thoracic vertebra in the LT muscle. The rib eye area (REA) at the 12th/13th rib interface was also measured. Subsequently, beef samples were collected and later sectioned into 2.54 cm steaks for physicochemical analyses. The first steak was taken between the 11th and 13th ribs and the others were in the cranio-caudal direction.

### 4.2. Chemical Composition of Meat

To evaluate the proximate composition, the samples were thawed in a refrigerator at 4 °C for 24 h and the subcutaneous fat was removed with the aid of a scalpel. The steak was then ground in a multiprocessor for five minutes, using approximately 180 g of sample [51]. The analyses were carried out by infrared spectroscopy in a FoodScan™ equipment (FOSS, Hillerød, Denmark), in which the average levels of moisture, protein, fat and total collagen were determined. The averages of moisture, protein, fat and ash were obtained through three readings per sample, and at each reading the sample was removed from the plate, homogenized again and returned to the plate for the next reading.

### 4.3. pH and Meat Color

The pH, instrumental color, cooking loss and shear force were measured according to the ageing periods (3, 10, and 17 days post-mortem). Ageing was carried out in a refrigerated BOD incubator (TE-371, TECNAL, Piracicaba, Brazil) at a temperature of 0 to 2 °C, in polyethylene packaging bags (20 × 30 cm; Bemis Company, São Paulo, Brazil) for high vacuum and low oxygen permeability. The pH was determined with a Hanna digital pH meter (Model HI 99163, Hanna Instruments, Woonsocket, RI, USA) equipped with a penetration electrode. Standard buffers (pH 4.0 and 7.0) were used in calibration procedures.

Meat color (L* = lightness; a* = redness; b* = yellowness) was obtained from the average value of three readings for each variable (L*, a* and b*), after 30 min of oxygenation. The CIELab system of the CR-400 colorimeter (light source A, absorbance angle 10°, display Y: 0.01% to 160% reflectance, Konica Minolta Sensing, Inc., Tokyo, Japan) was used. The colorimeter calibration was performed with a standard black and white plate.

### 4.4. Cooking Loss and Shear Force

To assess cooking loss (CL) and Warner-Bratzler shear force (WBSF), the procedure proposed by Wheeler et al. [52] was adopted and the recommendations of the American Meat Science Association were followed [53]. The samples were placed on a grid coupled to a glass refractory. A thermocouple connected to a digital thermometer (DT-612, ATP Instrumentation, Ashby-de-la-Zouch, England) was used, which was inserted in the center of each sample to monitor the internal end-point temperature.

The samples were cooked in an industrial electric oven (Feri90 Venâncio, Venâncio Aires, Rio Grande do Sul, Brazil) preheated to 170 °C and equipped with a thermostat to minimize temperature variations. Once the internal temperature of the steaks reached 40 °C, they were turned over and remained in the oven until the final temperature reached 71 °C. The samples were then kept at room temperature for 15 min, weighed, and refrigerated at 4 °C for 24 h. The CL was divided into evaporation loss (EL) and drip loss (DL), determined as percentage. The DL was obtained by weighing only the refractory before and after cooking the sample. The EL was obtained by weighing the sample before and after cooking.

For the determination of WBSF, eight cylinders with a diameter of 1.27 cm were sectioned in a Brookfield CT-3 Texture Analyzer (AMETEK Brookfield, Middleborough, MA, USA), equipped with a stainless steel 3.07-mm-thick Warner–Bratzler blade with a vee-shaped (60° angle) cutting edge. Results were reported as the average of eight values per sample, in Newton (N).

### 4.5. Proteomics

The extraction, precipitation, separation, imaging and protein identification procedures were carried out according to the literature [7,28], with minimal adaptations.

#### 4.5.1. Extraction and Precipitation of Proteins

Individual samples of LT muscle collected during the pre-rigor mortis period were individually processed to obtain two-dimensional electrophoresis gels (2D-PAGE). For each treatment (sex class), eight individual samples (biological replicates) were used (eight steer gels and eight heifer gels) with three technical replicates (three gels for each sample), totaling 48 gels. For each sample, approximately 0.2 g of the muscle was ground in 1.0 mL lysis buffer using an Ultra-Turrax high shear mixer (Marconi–MA102/E, Piracicaba, São Paulo, Brazil) at 20,000 rpm twice for 30 s. The protein extracts were separated from the solid part by 15 min of centrifugation at 10,000× *g* rpm at 4 °C. The protein content of these extracts was placed in 80% (*v*/*v*) acetone solution and kept at 5 °C for 2–3 h to ensure that the procedure occurred for a sufficient time. The centrifugation process was then repeated for 25 min at 10.000× *g* rpm to obtain protein pellets for quantification and 2D-PAGE. The obtained pellets were washed beforehand to quantify proteins and were used in electrophoretic runs. One portion of the protein pellets was re-solubilized in 0.5 M NaOH to quantify total protein. The total protein concentration of the bovine muscle tissue samples was quantified by the Biuret method [54].

#### 4.5.2. Protein Separation by Two-Dimensional Electrophoresis (2D-PAGE)

An additional protein fraction was re-solubilized in a specific buffer containing 0.07 M urea, 0.02 M thiourea, 2% 3-[(3-cholaminopropyl)-dimethylammonium]-1-propanesulfonate–CHAPS (*m/v*), 10% ampholyte (pH range 3–10), and 0.002% bromophenol blue. Moreover, 2.8 mg of 1,4-dithiothreitol was added and this solution was used in electrophoretic separations.

Briefly, approximately 375 μg of protein extracts (1.5 μg/μL) were loaded into first dimension strips (13 cm) and hydrated for 12 h. Subsequently, the protein extracts were separated on pH 3–10 with isoelectric focus in an Ettan IPGphor 3 device (GE Healthcare, United States), in which the proteins were fractionated by the isoelectric point, pH value when the net charge total protein is zero.

The strips were placed in equilibrium solutions for reduction, alkylation and weresubjected to the second dimension (2D) of electrophoresis in a 12.5% (*m/v*) polyacrylamide gel. At the end of the 2D run, approximately 500 mL of colloidal Coomassie stain was used to mark the protein spots of the gels for 72 h. These gels were subsequently destained with ultrapure water.

The gels were scanned, and the images were imported into ImageMaster Platinum software, version 7.0, for comparisons (contrasts) of images between treatments and obtaining information such as number of spots per gel, percentage of matching (correspondence between the spots proteins in the gels), isoelectric point (p*I*), molecular weight (MW), and volume of the spots. The correspondence (matching) of the gels within each sample (three technical repetitions) was greater than 95%, demonstrating that 95% of the spots were present in the technical replicates, indicating good reproducibility. For image comparisons, a reference gel per treatment was listed [55], which contained the highest number and best definition of spots, and the reference gel of a treatment was contrasted with each gel of another treatment, totaling 15 comparisons.

#### 4.5.3. Tryptic Digestion of Protein Spots and Identification of Proteins by ESI-MS/MS

Protein spots from experimental groups (heifers versus steers) were selected based on the molecular weight (MW) and isoelectric point (p*I*) obtained by image analysis and then cut out (fragments of approximately 1 mm^3^), and prepared according to the method of [56]. The sediments were transferred to microtubes and submitted to the following four steps: The first step involved the removal of the dye with 25 mM ammonium bicarbonate (Ambic)/acetonitrile (50:50, *v*/*v*) Step two was the reduction and alkylation in which the gel fragments were rehydrated in a reducing solution and incubated for 40 min at 56 °C.

After removal of the reducing solution, an alkylating solution was added and the fragments were incubated in the dark for 30 min at room temperature. Step three involved the digestion consisting of overnight incubation at 37 °C with 10 ng·μL^−1^ trypsin in 25 mM Ambic for 15 min (Trypsin Gold Mass Spectrometry, Promega, Madison, WI, United States). The final step saw the elution of peptides extracted from the gel using three steps: (A) 50% ACN with 1% formic acid incubated for 15 min at 40 °C under sonication, and the supernatant was collected and transferred to a new tube; (B) 60% methanol with 1% formic acid incubated for 15 min at 40 °C under sonication, and the supernatant was collected and transferred to a new tube; and (C) 100% ACN; the extracts were dried in a vacuum centrifuge and peptides were dissolved in 10 μL of 3% ACN with 0.1% formic acid.

The mass spectra of the peptides were obtained by analyzing aliquots of the solutions in a nanoACQUITY UPLC-Xevo TQ-MS System (Waters, Manchester, UK). The proteins were identified in the UniProt database (UniProtKB/Swiss-Prot database) for the *Bos taurus* genome.

#### 4.5.4. Bioinformatics

Bioinformatics analyses were conducted for the classification of differentially expressed proteins in muscle tissues from animals (heifers versus steers) in terms of biological processes (BP), molecular function (MF), and cellular components (CC). For this purpose, the accession number of the proteins identified by ESI/MS/MS were entered into the UniProt database (www.uniprot.org, accessed on 5 August 2022) and their FASTA sequences were extracted. After this step, the proteins were analyzed using the OMICSBOX v.2.0 (https://www.biobam.com/omicsbox/, accessed on 5 August 2022) and Blast2GO tools [57].

Additionally, the interactions between the proteins identified in the treatments were analyzed using the open source STRING 11.0 platform (https://string-db.org/, accessed on 20 August 2022). The same list of proteins whose expression differed between the experimental groups were used in these analyses [58].

Subsequently, further bioinformatics analyses were performed following the procedures described by Gagaoua et al. [7] using Metascape^®^ platform. Briefly, gene identifiers were converted using Uniprot Retrieve/ID mapping. Thus, key information regarding the proteins (gene names [GN]) and their relationships with carcass and meat quality traits described in the current study were annotated for each gender. These procedures aiming to compare the two protein lists to better understand the common and divergent molecular signatures. Hierarchical heatmap clustering was also created using enriched GO terms analyzed by Metascape^®^ (https://metascape.org/, accessed on 20 August 2022).

Additionally, the ProteQTL tool included in ProteINSIDE (http://www.proteinside.org/, accessed on 20 August 2022) was used for rapid searching of carcass and meat quality quantitive trait loci (QTL) among the list of putative biomarkers following the details described by Gagaoua et al. [59]. ProteQTL interrogates a public library of published QTL in the Animal QTL Database (https://www.animalgenome.org/QTLdb, accessed on 20 August 2022) that contains cattle QTL and association data curated from published scientific articles [7].

#### 4.5.5. Statistical Analysis

Carcass and meat quality variables were analyzed regarding the homogeneity and normality of the residues, which expressed by means and their respective errors. Data were submitted to analysis of variance (ANOVA) using the F Test and using the SAS GLM procedure (version 9.1, Cary, NC, USA). Comparisons between means were made using a Tukey’s Test and the *p* value < 0.05 was adopted as the critical level of probability [60,61,62]. The design was completely randomized according to the following model:

Yij = μ + ti + ϵij,

where, Yij is the observed value for the experimental unit referring to treatment i in repetition j; μ is the general effect of the mean; t is the treatment effect (gender) and ϵ is the experimental error.

The protein spot volumes data were imported into ImageMaster Platinum (v. 7.0) software and mean and standard deviation were calculated for selected spots. The images were compared between treatments by means of the matching of the spots regarding their distribution, volume, relative intensity, p*I*s and MW. The program generated an ANOVA within the comparisons between treatments, and the divergent protein spots (*p* < 0.05) were selected. Additionally, the Mann-Whitney test (Wilcoxon rank-sum test) was used when the normality criteria was violated in any of the treatments. For both tests (Student or Mann-Whitney), significance was detected at the 0.05 level. For all data, trends were considered at 0.05 < *p* ≤ 0.10.

## 5. Conclusions

The results obtained showed higher IMF content in meat from heifers compared to steers, with a better response to stress in the muscle of heifers. Even with immunocastration, males did not increase lipid synthesis in muscle tissue in the same proportion as females. The expression pattern of transport proteins, energy metabolism, cellular defense, and glycogenolysis differ among heifers and steers, suggesting greater oxidative capacity and lower glycolytic activity in the muscle of heifers. Such proteome changes in LT muscle help to explain the differences found in meat quality traits, particularly marbling.

## Figures and Tables

**Figure 4:**
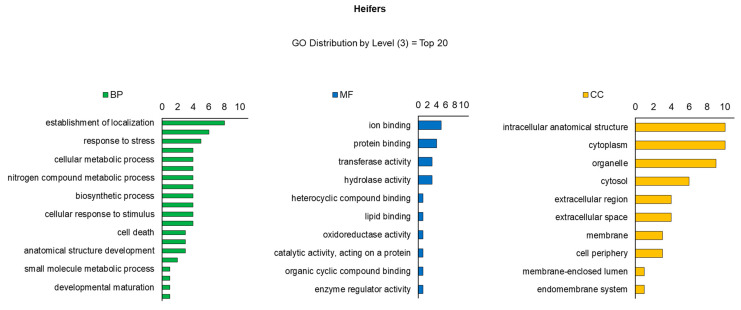
Classification of proteins identified in tissue samples (*Longissimus thoracis*) from immunocastrated F1 Montana-Nellore young steers feedlot finished. The OMICSBOX software was used to classify the proteins according to biological process (BP), molecular function (MF), and cellular component (CC).

**Table 1:** Live body weight and carcass traits of immunocastrated F1 Montana-Nellore young heifers and steers feedlot finished.

Variables ^1^	Heifers	Steers	SEM	*p*-Value
IBW (kg)	289.40	284.00	9.57	0.78
FBW (kg)	373.75	391.13	10.41	0.57
HCW (kg)	202.18	219.25	5.97	0.09
CY (%)	54.81	55.82	0.00	0.53
BFT (mm)	14.90	10.15	1.10	0.04
REA (cm^2^)	75.99	90.48	1.66	0.06

^1^ IBW and FBW: initial and final body weight, respectively; HCW: hot carcass weight; CY: Carcass yield; BFT: Backfat thickness; REA: Ribeye area.

**Table 2:** Meat quality traits of immunocastrated F1 Montana-Nellore young steers and heifers feedlot finished.

Variables ^1^	Heifers			Steers				*p*-Value	
	Ageing (Days)			Ageing (Days)			SEM	Gender	Ageing Time
	3	10	17	3	10	17			
L*	33.76	34.78	36.54	32.94	34.50	34.84	0.49	0.24	0.06
a*	16.26	16.55	16.73	15.94	16.73	15.99	0.14	0.45	0.53
b*	6.08	6.18	6.53	5.93	6.21	6.05	0.08	0.41	0.63
pH	5.60	5.71	5.73	5.60	5.68	5.75	0.02	0.65	<0.01
WBSF (N)	55.70	40.40	31.28	54.42	40.79	37.26	3.62	0.42	<0.01
EL (%)	24.27	20.43	17.10	21.46	17.47	17.14	0.01	0.01	<0.01
DL (%)	6.69	4.49	4.28	7.13	6.54	5.22	0.00	0.02	0.003
CL (%)	30.96	24.92	21.38	28.59	24.01	22.36	0.01	0.25	<0.01

^1^ L*: lightness, a*: redness, b*: yellowness, pH = meat pH, WBSF: Warner-Bratzler shear force, EL: evaporation loss, DP: drip loss, CL: cooking loss.

**Table 4:** List of the Quantitative trait loci (QTL) of carcass and meat quality traits and their chromosomes (Chr.) obtained using the list of the proteins from immunocastrated F1 Nellore-Montana young heifers and steers feedlot finished.

QTL Linked to QTLdb ^1^	Gene Symboles	UniProtID (Bovine)	Chr.
Marbling score (*n* = 4)	HSPA8; MYBPC1; PGM1; PYGM	A6QP89; P19120; P79334; Q08DP0	Chr.15; Chr.5; Chr.3; Chr.29
Fat thickness at the 12th rib (*n* = 4)	ATP5F1B; MB; MYBPC1; PGM1	P00829; P02192; A6QP89; Q08DP0	Chr.5; Chr.5; Chr.5; Chr.3
Intramuscular fat (*n* = 1)	ATP5F1B	P00829	Chr.5;
Juiciness (*n* = 2)	ENO1; PYGM	P79334; Q9XSJ4	Chr.16; Chr.29
Shear force (*n* = 5)	ALB; ALDOA; HSPA8; PEBP1; PYGM	P02769; A6QLL8; P19120; P13696; P79334	Chr.6; Chr.25; Chr.15; Chr.17; Chr.29
Tenderness score (*n* = 2)	ALDOA; PYGM	A6QLL8; P79334	Chr.25; Chr.29
Adhesion (*n* = 1)	TPM2	Q5KR48	Chr.8
Muscle pH (*n* = 1)	PKM	A5D984	Chr.10

^1^ ProteQTL tool included in ProteINSIDE (http://www.proteinside.org/, accessed on 20 August 2022) interrogates a public library of published QTL in the Animal QTL Database (https://www.animalgenome.org/QTLdb/, accessed on 20 August 2022) that contains cattle QTL and association data curated from published scientific articles.

## Data Availability

All relevant data are within the paper.

## Supplementary Materials

The following supporting information can be downloaded at: https://www.mdpi.com/article/10.3390/ijms232012259/s1.
