# Supplementary material for: Proteomics Unveils Post-Mortem Changes in Beef Muscle Proteins and Provides Insight into Variations in Meat Quality Traits of Crossbred Young Steers and Heifers Raised in Feedlot
Source: Int J Mol Sci. 2022 Oct 14;23(20):12259. doi: 10.3390/ijms232012259 (PMC9603352; doi:10.3390/ijms232012259)
Supplement: Supplementary file 1 [file ijms-23-12259-s001.zip › ijms-1963635-Supplementary Material.pdf]

Supplementary data

Table S1. Chemical composition of the *Longissimus thoracis* muscle of immunocastrated F1 Montana-Nellore young heifers and steers feedlot finished.

Variables	Heifers	Steers	SEM	*P*-value
Moisture (%)	72.46	74.07	0.26	<0.01
Protein (%)	22.30	22.52	0.10	0.32
Intramuscular fat (%)	4.32	2.64	0.29	0.002
Collagen (%)	1.22	1.20	0.02	0.77
